# Maternal Sepsis: The Diagnostic Challenge in a Comorbid Patient in Mexico

**DOI:** 10.7759/cureus.76695

**Published:** 2024-12-31

**Authors:** Yazmin Cárdenas Ramos, Floricel Olimpia Villegas Amador, Sandra Allison Barrueta Orive, Claudia Patricia Rojas Tapia, Jean Carlo Lopez Carrasco

**Affiliations:** 1 Medicine, Regional General Hospital 1 of Instituto Mexicano del Seguro Social (IMSS), Querétaro, MEX; 2 Transplant and Donation, Regional General Hospital 1 of Instituto Mexicano del Seguro Social (IMSS), Querétaro, MEX; 3 Internal Medicine, Regional General Hospital 1 of Instituto Mexicano del Seguro Social (IMSS), Querétaro, MEX; 4 Geriatrics, Regional General Hospital 1 of Instituto Mexicano del Seguro Social (IMSS), Querétaro, MEX

**Keywords:** diabetes gestational, maternal sepsis, pregnancy-related complications, sepsis treatment, urinary track infection

## Abstract

Maternal sepsis is a complication that can be difficult to diagnose in the early stages because symptoms can be vague or attributed to other conditions. We present the case of a 38-year-old woman, in her third pregnancy with a diagnosis of uncontrolled gestational diabetes, who developed urinary origin sepsis in the second trimester of pregnancy. The patient was initially admitted with a clinical presentation interpreted as a urinary tract infection. However, her condition quickly deteriorated with sudden dyspnea, hypotension, and tachycardia. She was approached as a probable aortic dissection and transferred to a tertiary care facility for definitive treatment. Upon arrival at the reference center, the diagnosis of urinary focus sepsis was established, with imaging studies showing right pyelocaliceal dilatation and inflammatory process. The appropriate antibiotic treatment was delayed due to the lack of an early diagnosis, leading to clinical deterioration that necessitated urgent surgery. The therapeutic approach included broad-spectrum antibiotics and the placement of a right JJ catheter with drainage of purulent material, followed by intensive management. This case highlights the diagnostic challenge posed by maternal sepsis and underscores the critical role of early recognition through scoring systems, emphasizing the need for increased clinical suspicion and immediate attention.

## Introduction

The proposed concept of maternal sepsis is "a potentially fatal condition defined as organ dysfunction caused by an infection during pregnancy, childbirth, the puerperium, or after an abortion" [1], which has been considered an unregulated hyperinflammatory phase mediated by cytokines related to the stimulation of innate or adaptive immunity [2]. Over the last 15 years, the definition of sepsis in pregnancy has varied significantly; however, one aspect concerning this study population is the increased incidence of septic shock associated with multimorbidity. 

Although septic shock is rare during pregnancy, it leads to a high rate of premature deliveries [3], and can cause perinatal death and maternal reproductive damage in case of survival. The reported maternal mortality in pregnant women with severe sepsis is 7-8%, 20-40% with organ dysfunction, and increases to 60% if shock occurs. Approximately 20% of these will have intrauterine fetal death [4]. In first-world countries, 23% of all maternal deaths were related to sepsis [5]. In Mexico, according to data from the National Institute of Statistics and Geography (INEGI) in 2023, sepsis ranks third among causes of maternal mortality, representing 7.8% of maternal death cases [6].

The identification of sepsis requires the recognition of infection and acute organ dysfunction. Pregnancy represents an immunocompromised state created not to reject the growing fetus, which predisposes the mother to infection [7]. Gestational diabetes is the most common metabolic disorder of pregnancy, known as a disease that increases the possibility of infections [8]. Additionally, there are several risk factors for maternal sepsis, independent of gestational diabetes, such as cervicovaginitis, colonization by group B Streptococcus, premature rupture of membranes, cerclage, vaginal trauma, obesity, immune disorders, and the most common cause of sepsis in pregnant women is urinary origin; the most common clinical presentation being pyelonephritis, and like in non-pregnant populations, the etiological agent is *Escherichia coli* [9].

There is a scoring system to evaluate the severity of organ dysfunction called Quick Sepsis-Related Organ Failure Assessment (qSOFA). It is based on two abnormalities out of the three parameters that could be evaluated at the patient's bedside; however, these are less applicable in the obstetric context since respiratory rate, partial pressure of carbon dioxide (PaCO2), heart rate, and white blood cell count during a normal pregnancy can meet systemic inflammatory response syndrome (SIRS) criteria. Hence, the obstetric q-SOFA has been adapted to increase sensitivity (from 37.5% to 81.2%) and specificity (from 72.2% to 75%) in pregnant patients [10]. However, the qSOFA system is a poor screening tool for predicting severe maternal morbidity in pregnant patients with infections [11]. Although there are multiple scoring systems for use with obstetric patients, they also have numerous controversies. An efficient scoring system can be a valuable tool to reduce maternal morbidity and mortality by helping in the timely identification of high-risk sepsis patients, thus intensifying their management [12], since in most studies, pregnancy has been an exclusion criterion from major sepsis trials to date, leaving clinical decisions up to the treating physician's preference [13]. It is crucial to initiate treatment promptly, as delay in antibiotic administration can have severe consequences such as a notable increase in mortality, highlighting the importance of timely and effective intervention.

Mexico, being a developing country with limited availability of diagnostic tools, must be even more meticulous regarding clinical suspicion and approach to a pregnant patient admitted to the emergency service; thus, this report aims to analyze the approach, identification, and management of obstetric septic shock in a comorbid pregnancy in the second trimester.

## Case presentation

A 38-year-old female patient presented with a 23.1-week pregnancy plus uncontrolled gestational diabetes, and with signs of abdominal pain syndrome, transfixing type, intensity 9/10, radiating to the ipsilateral inguinal region. She had a past history of closed-angle glaucoma diagnosed three years ago with unspecified treatment, and laparoscopic cholecystectomy three years ago. Gestational diabetes was diagnosed before 20 weeks of gestation. Her gynecologic-obstetric history included two previous pregnancies (one abortion seven years ago and one cesarean five years ago), and regular menstrual cycles, with no history of preeclampsia, eclampsia, or gestational diabetes in previous pregnancies.

She was evaluated by Gynecology, and management for complicated urinary tract infection was initiated, empiric treatment based on quinolones without the results of the urine culture, with a favorable clinical response. However, one day after admission, she presented with sudden onset dyspnea associated with diaphoresis with exacerbation recorded by oximetry at 80% and a heart rate of 150 bpm, fever of 38°C, hemoglobin 8.7 mg/dL, and D-dimer 5000 ng/ml. An approach to rule out pulmonary thromboembolism (TEP) was started with angio-CT, where a probable aortic dissection in the ascending portion classified as Stanford A Debakey II was reported. Her transfer to a tertiary care level was requested.

**Figure 1:**
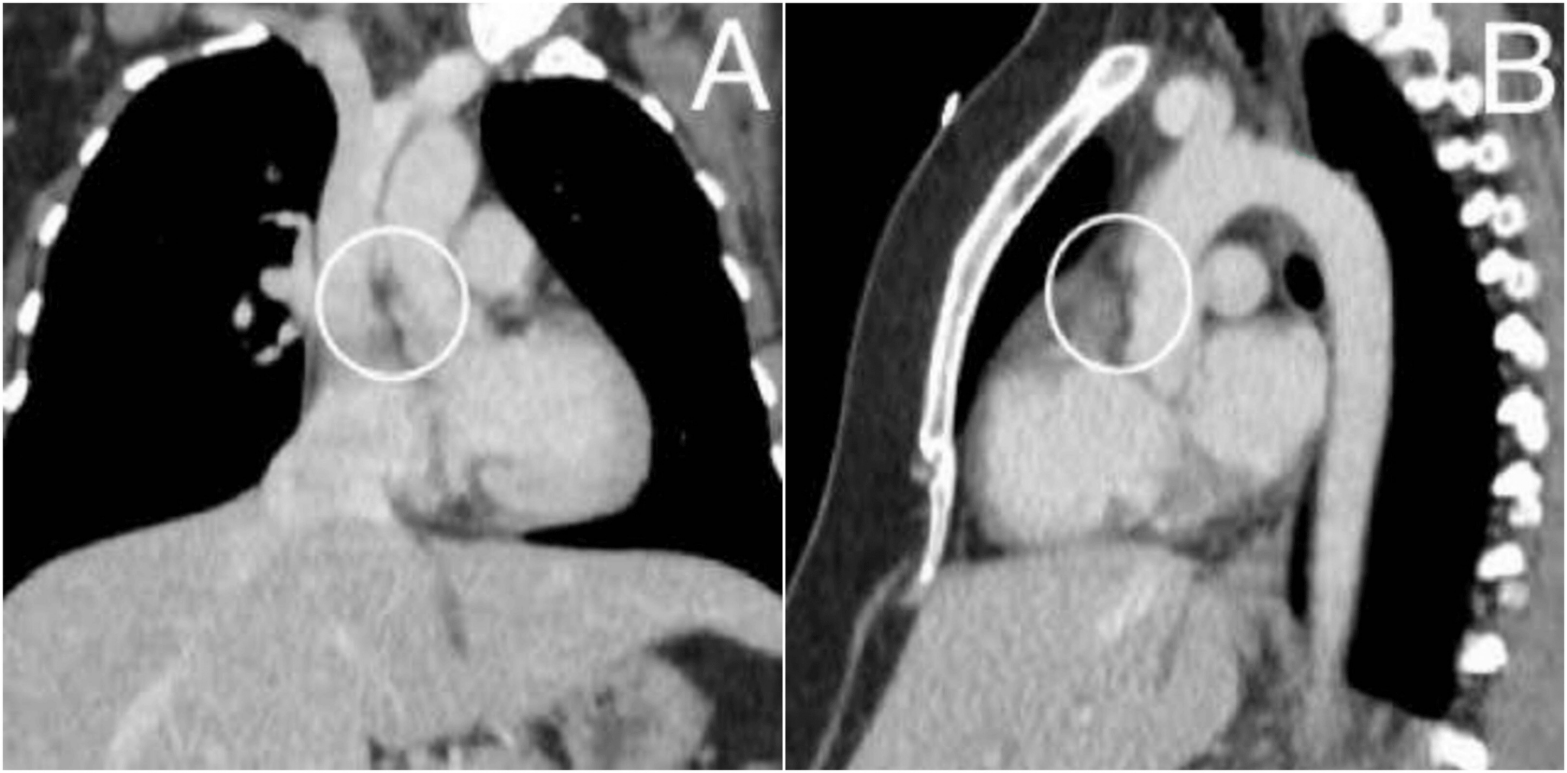
Thoracic Aortic Angio-CT coronal (A) and sagittal (B) slices showing no findings suggesting discontinuity of the intimal membrane at the aortic lumen level, nor filling defects or indirect findings suggesting pulmonary segmental branch thromboembolism, which translates into an absence of apparent vascular compromise. As an incidental finding, a hypodense, non-continuous line at the vascular lumen level is observed, not following typical trajectories, parallel to the acquisition plane, suggesting an artifact due to reconstruction or flow movement.

**Figure 2:**
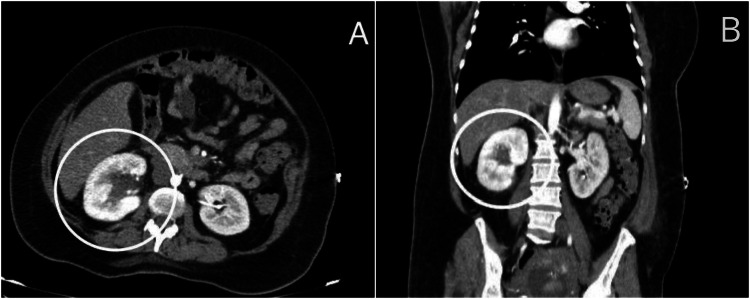
Renal Angio-CT, arterial phase, axial (A) and coronal (B) slices, showng a delay in the nephrogram of the left kidney compared to the right kidney, compatible with contralateral compensatory phenomenon due to the inflammatory process in the right kidney. Right kidney pyelocaliceal dilatation, evident in both slices, which may be related to an obstructive process or complicated infection, perirenal fat streaking characteristic of changes consistent with an inflammatory process.

She was received at the reference center with signs and laboratory tests of sepsis (Table 1), for which third-generation cephalosporin was indicated. The angio-CT was reinterpreted where aortic dissection was ruled out (Figure 1); however, right pyelocaliceal dilatation and inflammatory process were evident (Figure 2). Still awaiting the culture result, the antibiotic management was escalated to carbapenems. Subsequently, she was evaluated by Gynecology where a normal evolving pregnancy was reported without criteria for blood transfusion. At the same time, she was inter-consulted with the Urology service, who proceeded with the placement of a right JJ catheter with drainage of purulent material. Post surgery, she was admitted to the intensive care unit (ICU) due to hemodynamic instability and the need for mechanical ventilation. She was evaluated by infectious diseases due to the presence of septic shock from a urinary focus and pulmonary tracking with the presence of basal consolidation in the left hemithorax with probable intra-hospital pneumonia, for which it was decided to adjust previous antibiotic management by adding oxazolidinones.

**Table 1:** Complete Blood Count

Parameters	Day 1	Day 2	Day 3	Reference range
White blood cell count (K/uL)	12.5	3.1	8.66	4 - 11
Neutrophile (%)	86.7	83.1	62.1	37- 75
Hemoglobin (g/dl)	8.7	10.3	9.6	11- 18
Hematocrit (%)	12.5	31.2	27.8	36- 56
Platelet counts (K/uL)	209	132	54	150 - 450

During her inpatient follow-up, metabolic acidosis with an elevated anion gap was documented on multiple occasions, ketonuria but with blood glucose levels below 200 mg/dL, integrating the diagnosis of euglycemic diabetic ketoacidosis, managed by the ICU based on hydration, potassium replacement, and insulin infusion pump until meeting resolution criteria. The patient evolved favorably, weaning off invasive mechanical ventilation and insulin pump after one day in the unit, fulfilling resolution criteria, and thus initiating a basal insulin regimen. She was discharged from tertiary care to a regional hospital, where it was decided to continue close monitoring in the ICU for urosepsis, with plans to complete a broad-spectrum antibiotic regimen and therapeutic management. Over the following days, the patient showed a tendency towards improvement; however, she did not complete treatment and decided on voluntary discharge despite explanations regarding her health status and delicate prognosis.

## Discussion

Early recognition of sepsis in pregnant patients is crucial to reducing morbidity and mortality. Although pregnancy introduces physiological changes that may obscure the presentation of sepsis, treatment should follow the same principles as in nonpregnant populations. The Surviving Sepsis Campaign protocols still recommend early recognition, timely administration of broad-spectrum antibiotics, and fluid resuscitation within the first hour of recognition, even in pregnant or postpartum patients with septic shock or a high probability of sepsis [14]. Empiric therapy should include broad-spectrum β-lactams, such as third-generation cephalosporins, possibly combined with aminoglycosides like gentamicin until culture results are available to tailor therapy [15].

In the case of the current patient, it was particularly important to suspect complicated infections, especially in the presence of diabetes, which increases susceptibility to maternal sepsis. However, normal physiology of pregnancy can mask signs of sepsis, delaying diagnosis. During the patient’s hospital stay, laboratory findings revealed progressive clinical deterioration consistent with SIRS. However, the lack of a single gold-standard diagnostic test highlights the ongoing challenge of diagnosing sepsis easily and accurately. 

According to the American College of Obstetricians and Gynecologists (ACOG), urinary tract infections (UTIs) are the most common infection among pregnant women and have been associated with maternal and fetal complications [16]. UTI should be suspected based on the presence of symptoms that may be supported by a positive urinalysis result and is confirmed by the gold standard: the urine culture. A urine culture would have been useful to confirm the diagnosis; however, the cost is high and the waiting time for the results is very long, so it is emphasized that clinical suspicion and the approach according to the signs and symptoms presented in the patient are the key to reach a diagnosis and start empirical therapy in the first hour of recognition as indicated by the Guidelines on Surviving Sepsis Campaign [17].

Well-adapted and standardized criteria are needed to facilitate diagnosis and prevent adverse outcomes in women with maternal sepsis [18]. The obstetric qSOFA criteria use clinical data (blood pressure, respiratory rate, and mental status) that allow for rapid diagnosis; however, concerns have been raised that altered mental status is not a common presenting symptom for maternal sepsis, potentially making it less useful in the obstetric population [19]. Among the scores evaluated, SIRS has the highest sensitivity (0.93) and qSOFA has the highest specificity (0.95) for diagnosing sepsis. However, SIRS is less specific (0.63) and qSOFA is less sensitive (0.5). The sensitivity and specificity of the National Early Warning Score (MEW) are 0.82 and 0.87, respectively [20]. The California Maternal Quality Care Collaborative (CMQCC) and the United Kingdom Obstetric Surveillance System (UKOSS) criteria have shown lower false-positive rates and high sensitivity, making them effective for use during delivery admissions [21]. On the other hand, biomarkers such as procalcitonin and C-reactive protein (CRP) have been studied for their diagnostic performance in maternal sepsis. Procalcitonin has shown the highest area under the curve, indicating its potential utility as an adjunct in the clinical diagnosis of maternal sepsis [22].

Regarding differential diagnoses, when there is suspicion of aortic dissection due to sudden onset of dyspnea, it occurs in four to five per million pregnancies, and despite its low incidence, it's the third most common cause of maternal cardiovascular death, and one must keep in mind that this condition associated with pregnancy can have catastrophic consequences for both mother and fetus [23]. It's also worth noting that according to the patient's anamnesis in the present case, no identifiable risk factors were present that would support the suspicion of this condition, which underscores the importance of clinical judgment and imaging findings supporting an artifact rather than true vascular pathology, where the hypodense linear trace at the vascular lumen level not following a linear path or being discontinuous suggests an acquisition artifact or flow movement in the performed angio-CT. This highlights the importance of integrating clinical judgment with imaging to avoid unnecessary interventions.

While the management of maternal sepsis aligns with established principles for nonpregnant populations, obstetric-specific considerations are critical for timely diagnosis and treatment. There is still a need to improve and correctly use tools to detect early organ dysfunction before they have fatal repercussions for the pregnant woman and the fetus.

## Conclusions

The diagnosis of maternal sepsis remains a medical challenge for second-level hospitals. In this case, it is evident that pregnant women with underlying comorbidities, who are experiencing uncontrolled carbohydrate metabolism, combined with their physiological state inherent to pregnancy which conditions a decreased immunity, can foster the development of infections leading to septic shock. Therefore, timely detection of signs and symptoms is crucial in suspecting maternal sepsis, as this condition can rapidly progress to significant clinical deterioration if not treated early and intensively. It is thus recommended to initiate antibiotic therapy immediately, adjusting as necessary based on clinical evolution and microbiological study results. Although urine and blood cultures are standard procedures for diagnosing maternal sepsis, their impact on modifying antibiotic treatment is limited. In less developed countries like Mexico, the hospital's therapeutic decisions are often based more on the patient's clinical progress.

The prioritization of creating and validating tools that allow for the development of clear and standardized diagnostic criteria for maternal sepsis and septic shock, tailored to the changes inherent to pregnancy, corresponds to highly effective strategies to reduce the impact of these conditions on maternal health worldwide. Effective prevention, early recognition, and appropriate treatment of maternal infections and sepsis can help reduce the burden of sepsis as an underlying and contributing cause of morbidity and mortality.
